# Changes in the disease burden of breast cancer along with attributable risk factors in China from 1990 to 2019 and its projections: An analysis of the global burden of disease study 2019

**DOI:** 10.1002/cam4.5006

**Published:** 2022-07-03

**Authors:** Jie Li, Cui Chen, Jinjin Nie, Lili Wang, Zhen Zhang, Yuli Li

**Affiliations:** ^1^ Dongying People's Hospital Dongying People's Republic of China; ^2^ Dongying Center for Disease Control and Prevention Dongying People's Republic of China

**Keywords:** breast cancer, disease burden, risk factor, spatiotemporal trend

## Abstract

**Background:**

To investigate the secular trends in breast cancer burden with attributable risk factors, and make projections over time, which would contribute to the control and prevention of breast cancer.

**Methods:**

We extracted detailed data on breast cancer incident cases and age‐standardized incidence rate (ASIR), deaths and age‐standardized mortality rate (ASMR), disability‐adjusted life‐years (DALYs), and age‐standardized DALYs rate (ASDR), as well as the attributable risk factors in China from the Global Burden of Diseases Study 2019. The estimated annual percentage change (EAPC) was calculated to quantify the changing trends. The national DALYs attributable to Socio‐demographic Index (SDI) values were also presented. Projections to 2030 were estimated using the Bayesian age‐period‐cohort model.

**Results:**

From 1990 to 2019, the number of breast cancer incident cases increased fourfold to 375,484, with deaths and DALYs over doubling to 96.306 and 2,957,454, respectively. The ASIR (EAPC = 2.84; 95% CI, 2.74–2.95) and ASMR (EAPC = 0.06; 95% CI, 0.00–0.12) increased, while the ASDR decreased with the EAPC of −0.13 (95% CI, −0.19 to −0.06) at the same period. The ASDR varied across provincial regions, which appeared to be in a wave‐like upcurve with SDI values increasing. High body mass index became the first contribution to breast cancer DALYs for females in 2019, and alcohol use for males. Breast Cancer incident cases and deaths would increase to 587.7 and 125.6 thousand in 2030, of which there will be 577.1 and 122.7 thousand for females, and 10.6 and 2.9 thousand for males, respectively.

**Conclusion:**

Breast cancer remains a major public health problem in China. The absolute burden has been increasing over time, and varied across sex and regions. To control the potential risk factors and develop specific strategies will help to reduce the disease burden.

## Graphical abstract

The study aimed to describe the spatiotemporal trends in breast cancer burden along with the attributable risk factors in China. From 1990 to 2019, the national number of breast cancer incident cases increased fourfold to 375.5 thousand, and the number of deaths and DALYs over doubled to 96.3 thousand and 2957.5 thousand, respectively. The projected numbers will continue to increase until 2030 for both sexes. High body mass index became the first contribution to breast cancer DALYs for females in 2019, and alcohol use for males.

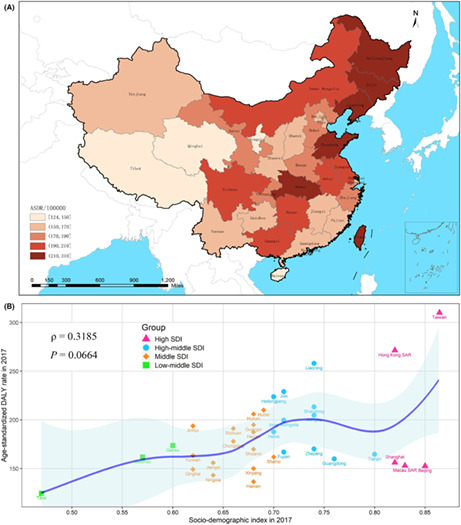

## 1 INTRODUCTION

Breast cancer is one of the most burdensome malignancy among females around the world,^1^ the cases and deaths of which accounts for approximately 25% of all diagnosed cancers and 15% of cancer‐associated deaths.^2^ Although the rates of female breast cancer incidence and mortality in China were lower than that in Europe, Oceania or Northern America, nearly 20% of global cases and deaths occurred in China due to the largest human population worldwide.^2^, ^3^ On the other hand, male breast cancer was usually neglected in previous studies, for the relatively low incidence rates and managed following the recommendations for postmenopausal women,^3^, ^4^, ^5^ while the molecular and clinicopathologic characteristics of male breast cancer were different from female breast cancer.^6^

Various risk factors have been established for breast cancer, including some hereditary factors,^7^ and nonhereditary risk factors, such as high body mass index (BMI),^8^ poor socio‐economic status,^9^ post‐pregnancy^10^ and hormone‐replacement therapy.^11^ These modifiable risk factors varied with region and time in China, for this country underwent a massive social transformation in the past decades, leading to spatiotemporal variations in breast cancer burden. Although the previous global burden of breast cancer has been described by several studies,^12^, ^13^, ^14^ the current and potential trends in breast cancer burden attributable to risk factors among females and males in China are still unclear, which will be the foundations of the subsequent measures to control and prevent cancer.^15^, ^16^

The Global Burden of Disease Study (GBD) 2019 provides the annual estimates of health loss from 369 diseases and injuries, and associated risk factors in 204 countries and territories from 1990 to 2019.^17^ The GBD 2019 develops some online systems to make all the data easily available and accessible, so that the researchers could conveniently use these data to analyze the changing trends in different diseases. Based on the data of GBD 2019, we aim to offer the newest insights into the breast cancer burden along with the attributable risk factors in China by sex and age from 1990 to 2019, and make projections until 2030, which is beneficial to guiding resource allocation and strategies development, as well as the precise control and prevention of breast cancer.

## 2 MATERIALS AND METHODS

### 2.1 Study data

In GBD 2019, breast cancer was defined as the ICD‐10 code C50‐C50.9, D05‐D05.9, D24‐D24.9, D48.6, and D49.3, and the ICD‐9 code 174–175.9, 217–217.8, 233.0, 238.3, 239.3, and 610–610.9.^17^ Data on breast cancer incident cases, deaths, disability‐adjusted life‐years (DALYs) and respective age‐standardized rates (ASIR, ASMR, and ASDR) in China from 1990 to 2019 were retrieved by each sex and age group (5‐year age groups from 15–94 years, and the group of 95+ years) from the online Global Health Data Exchange query tool (https://vizhub.healthdata.org/gbd‐results/), where the specific filter rules were predefined, including “China” for “Location”, “Breast cancer” for “Cause”, “1990–2019” for “Year”, and “Incidence, Deaths, DALYs” for “Measure”. They were reported as the annual estimates with its 95% uncertainty intervals (*UI*s), which were determined using the 2.5th and 97.5th centiles for a parameter estimate of the ordered 1000 draws.

### 2.2 Estimation framework

The whole estimation framework began with cancer mortality.^18^ The breast cancer mortality data in China were assembled from surveillance data of the China Disease Surveillance Points system, vital registration data of the China Cancer Registry and the Chinese Center for Disease Control and Prevention, literature reviews, and national surveys, which must be standardized first and imported into the Cause of Death Ensemble Model, to generate estimates of cancer‐specific mortality by age, sex and year. Then, the mortality‐to‐incidence ratio (MIR) was generated based on the population‐based cancer registries that provided both incidence and mortality of breast cancer. Incidence was calculated by the breast cancer mortality divided by the estimated MIRs.

Breast cancer DALYs were the sum of years of life lost and years lived with disability. On one hand, years of life lost were calculated as the product of estimated breast cancer deaths and the standard life expectancy at age of death. On the other hand, SEER mortality, incidence, and survival data were obtained from the SEER*Stat Databases to establish models of correlation between the relative survival and the SEER MIRs using Poisson Regression. Then these models were applied to the GBD data to calculate the relative survival data, which were further adjusted for the background mortality to calculate the absolute survival data. The prevalence was estimated by combining the survival data with the GBD incidence estimates, and divided into four sequelae: Diagnosis/treatment, remission, metastatic/disseminated, and terminal phase. Years lived with disability were calculated by multiplying the prevalence of each sequela and the disability weights for the health state associated with this sequela, and by adding the complications caused by breast cancer treatment.

Finally, the GBD world population age standard was used for the age standardization. An updated age‐standardized population structure was generated using the non‐weighted mean of 2019 age‐specific proportional distributions of all countries/regions with a population >5 million people in the GBD 2019 population estimates.^17^ The detailed input sources were available via online data source tools (http://ghdx.healthdata.org/gbd‐2019/data‐input‐sources).

### 2.3 Geographical units and Socio‐demographic Index (SDI)

Detailed subnational estimates of breast cancer burden were not provided for China by GBD 2019, while a relevant study estimated the disease burden metrics at the province level in China using the GBD methods,^19^ where we extracted data on breast cancer DALYs and SDI values of 34 provincial administrative units (excluding Xinjiang Production and Construction Corps) in 2017, which consisted of 23 provinces, five autonomous regions, four municipalities, Hong Kong Special Administrative Regions (SAR), and Macao SAR. SDI, a composite indicator of development status strongly correlated with health outcomes developed by GBD collaborators, which was the geometric mean of total fertility rate under age 25 years, average education for those with age 15 years or older, and lag distributed income per capita, ranging from 0 (the lowest income, lowest educational attainment level and highest fertility) to 1 (the highest income, highest educational attainment level and lowest fertility),^20^ was used to divide the provinces into five SDI groups (high, high‐middle, middle, low‐middle, and low) based on quintiles.

### 2.4 Attributable burden

The attributable burden of breast cancer was estimated by the comparative risk assessment framework, which contained six steps: Inclusion of risk‐outcome pairs; estimation of relative risks (RRs) as a function of exposure; estimation of exposure levels and distributions; determination of the counterfactual level of exposure; determination of the theoretical minimum risk exposure level; computation of population attributable fractions (PAF) and attributable burden; and estimation of mediation of different risk factors through other risk factors.^21^ The risk factors for breast cancer were identified based on the World Cancer Research Fund grades of convincing or probable evidence, finally including high fasting plasma glucose, alcohol use, high BMI, diet high in red meat, low physical activity, and tobacco (smoking, chewing tobacco, and secondhand smoke). Then, the RRs, which were derived from previous primary studies or secondary meta‐analyses, and had been adjusted for the potential confounders, were used to calculate the PAFs, and further to calculate the attributable burden for each risk‐outcome pair. To describe the trends in each risk‐outcome pair, we obtained the attributable breast cancer DALYs in China by sex, year, and age.

### 2.5 Statistical analyses

We showed the secular trend in breast cancer burden along with its attributable risk factors by sex, year, and age. The estimated annual percentage change (EAPC) was introduced to measure the temporal trends in age‐standardized rates (ASRs). A regression line, *y* = *α* + *βx* + *ɛ*, was fitted to the natural logarithm of the rates, where *y* referred to ln(ASR), and *x* the calendar year. EAPC was calculated as 100 × (exp[*β*] − 1) and its 95% confidence intervals (*CI*s) were obtained from the linear model.^22^ The rates would be upward if the EAPC and the corresponding 95% *CI* were positive, downward if they were negative, and otherwise stable. Moreover, to explore the average annual percentage change of breast cancer ASRs in China from 1990 to 2019, we conducted the Joinpoint Regression analysis, for this model could provide visualized and interpretable results. Joinpoint Regression uses a piecewise linear‐regression to estimate the adaptive trends by one or more line‐segments,^23^ which can characterize the whole changes of the data and identify the timepoint when changes are significant. At the provincial level, we mapped the geographical trend in ASDR, and explored the adapted curvilinear relation between ASDR and SDI values, using Locally Estimated Scatterplot Smoothing Regression, which was a nonparametric technique and could reveal trends in data that might be difficult to model using a parametric curve.

Projected numbers and ASRs of incidence and mortality until 2030 were extrapolated from the Bayesian age‐period‐cohort (BAPC) model. The BAPC model turned out to be the most appropriate statistical methods of projecting the cancer burden compared with generalized additive model, smooth spline model, Nordpred model, Joinpoint model, and Poisson regression, especially for short‐term projections.^15^, ^24^, ^25^ Briefly, the age‐period‐cohort model, *η*_*ij*_ = log(*λ*_*ij*_) = *μ* + *α*_*i*_ + *β*_*j*_ + *γ*_*k*_, was fitted as a log‐linear Poisson model, in which *λ*_*ij*_ indicated the number of cases, *μ* was the intercept, and *α*_*i*_, *β*_*j*_, *γ*_*k*_ were age, birth cohort and the period in which the event occurred, respectively.^24^ *i* (1 ≤ *i* ≤ *I*) indicated age group at time *j* (1 ≤ *j* ≤ *J*), and the birth cohort index *k*, *k* = *j* + *M*(*I* − *i*), depended on the age group and period index, as well as the width of age groups and period intervals, where *M* indicated the width of age groups (5 in this analysis) divided by the period intervals. BAPC model (the BAPC package via software R) fitted with Integrated Nested Laplace Approximation (the INLA package via software R) was used to project breast cancer burden by sex until 2030, based on the hypothesis of inverse‐gamma prior distribution of the GBD 2019 data we retrieved, including age, period, and cohort effects (via the second‐order random walk model) to adjust for excessive dispersion. The reference in 2020–2030 was calculated with the positive side as increasing by 1% every year and the negative side as decreasing by 1% every year based on the observed rates in 2019. All results were visualized by ggplot2 and RcolorBrewer packages via software R (Version 4.1.3).

## 3 RESULTS

### 3.1 The temporal trend in breast cancer burden in China

**FIGURE 1:**
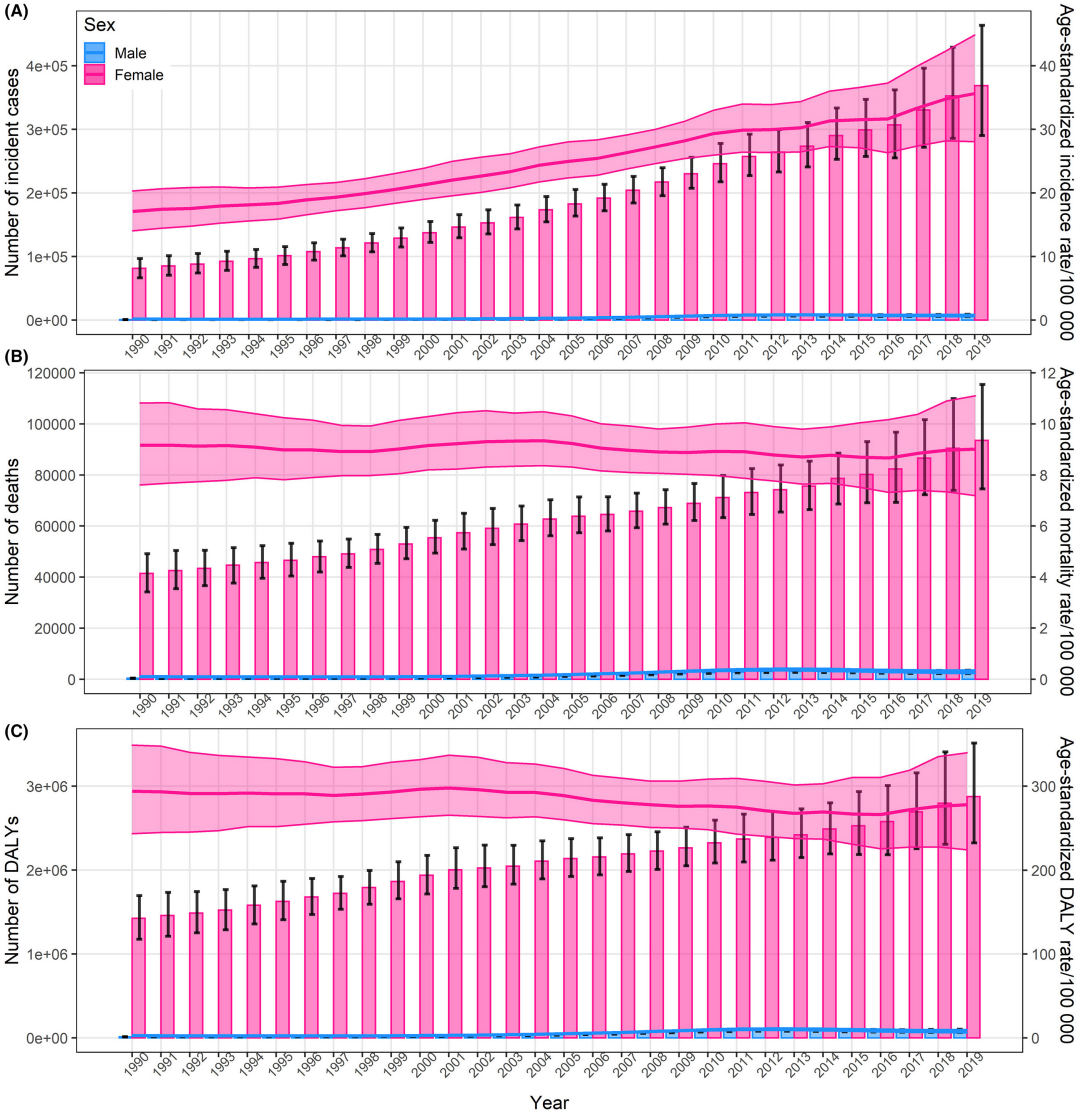
The temporal trend in number and ASRs of breast cancer incidence (A), mortality (B), and DALYs (C) by sex in China, 1990–2019. The bar graphs represent the observed number and the error bars indicate the 95% uncertainty intervals (*UI*s); the line charts represent the ASRs and the shading indicates the 95% *UI*s. ASR, age‐standardized rate; DALYs, disability‐adjusted life‐years. The male information is also separately shown in Supplementary Materials.

In China, a total of 375,484 breast cancer incident cases (95% *UI*: 296,626, 469,983), 96,306 deaths (95% *UI*: 77,323, 118,090) and 2,957,454 DALYs (95% *UI*: 2,408,511, 3,590,166) occurred in 2019. From 1990 to 2019, the number of incident cases increased fourfold, and the ASIR increased with an EAPC of 2.84 (95% *CI*: 2.74, 2.95). Meanwhile, the number of deaths and DALYs over doubled, but ASMR and ASDR had little change, with an EAPC of 0.06 and −0.13 (Tables 1, 2, 3). Figure 1 showed the different temporal trends in breast cancer burden in females and males in China. Females had far more disease burden than males, but males had larger temporal changes in ASIR, ASMR, and ASDR than females, with the EAPC of 8.74, 6.21, 6.59 in males and 2.70, −0.14, −0.35 in females, respectively (Figure 1; Tables 1, 2, 3). The results of Joinpoint Regression presented that the patterns in ASRs of breast cancer in China from 1990 to 2019 varied with time, but the overall trends in ASRs of breast cancer were almost monotonous (Figures S1–S3).

**TABLE 1:** The number of incident cases and the age‐standardized incidence rates of breast cancer in China in 1990 and 2019, and the estimated annual percentage changes from 1990 to 2019

Characteristics	1990		2019		1990–2019
	Incident cases^a^	ASIR^b^	Incident cases^a^	ASIR^b^	EAPC in ASIR^c^
Overall	81.62 (66.87, 97.10)	8.54 (7.07, 10.11)	375.48 (296.63, 469.98)	18.32 (14.50, 22.93)	2.84 (2.74, 2.95)
Sex					
Male	0.55 (0.45, 0.66)	0.13 (0.10, 0.15)	7.11 (5.34, 9.08)	0.69 (0.53, 0.88)	8.74 (7.59, 9.89)
Female	81.07 (66.34, 96.52)	17.07 (14.02, 20.30)	368.37 (290.09, 463.34)	35.61 (28.07, 44.81)	2.70 (2.60, 2.79)
Age group					
15–19	0.26 (0.21, 0.31)	0.20 (0.17, 0.24)	0.26 (0.21, 0.31)	0.34 (0.28, 0.41)	1.56 (1.32, 1.80)
20–24	0.58 (0.45, 0.73)	0.43 (0.34, 0.55)	0.78 (0.60, 1.02)	0.96 (0.73, 1.25)	2.30 (1.80, 2.80)
25–29	1.28 (0.99, 1.65)	1.16 (0.90, 1.50)	3.42 (2.53, 4.48)	3.09 (2.29, 4.04)	3.16 (2.46, 3.85)
30–34	3.15 (2.48, 3.94)	3.56 (2.80, 4.45)	10.96 (8.36, 13.98)	8.49 (6.48, 10.83)	2.59 (2.01, 3.17)
35–39	7.81 (6.13, 9.69)	8.53 (6.70, 10.59)	17.15 (12.84, 21.92)	17.00 (12.73, 21.73)	2.20 (2.07, 2.33)
40–44	9.74 (7.78, 11.90)	14.49 (11.56, 17.70)	30.29 (23.26, 38.64)	29.80 (22.89, 38.01)	2.43 (2.35, 2.52)
45–49	9.91 (7.84, 12.04)	19.17 (15.17, 23.27)	44.97 (34.55, 56.76)	37.05 (28.47, 46.77)	2.01 (1.63, 2.4)
50–54	11.36 (9.19, 13.81)	23.77 (19.22, 28.89)	59.14 (45.77, 74.63)	47.27 (36.59, 59.65)	2.65 (2.43, 2.88)
55–59	11.81 (9.61, 14.23)	27.17 (22.12, 32.74)	52.87 (41.10, 66.89)	55.75 (43.33, 70.53)	3.2 (2.72, 3.69)
60–64	9.06 (7.47, 10.72)	25.57 (21.10, 30.26)	47.9 (38.01, 59.23)	60.98 (48.39, 75.40)	3.62 (3.31, 3.93)
65–69	6.94 (5.86, 8.20)	25.36 (21.4, 29.97)	45.72 (37.13, 56.42)	64.96 (52.76, 80.16)	3.71 (3.47, 3.95)
70–74	4.61 (3.93, 5.44)	24.46 (20.82, 28.85)	28.68 (23.54, 35.19)	59.92 (49.18, 73.53)	3.41 (3.26, 3.57)
75–79	2.85 (2.43, 3.29)	24.94 (21.31, 28.85)	16.93 (13.86, 20.61)	56.73 (46.44, 69.04)	3.17 (2.98, 3.36)
80–84	1.54 (1.32, 1.79)	27.32 (23.46, 31.81)	10.59 (8.47, 12.81)	55.52 (44.41, 67.18)	2.76 (2.57, 2.94)
85–89	0.57 (0.49, 0.68)	29.97 (25.41, 35.30)	4.23 (3.30, 5.14)	49.77 (38.80, 60.47)	1.83 (1.73, 1.94)
90–94	0.13 (0.10, 0.15)	34.34 (28.11, 40.24)	1.28 (0.99, 1.54)	56.91 (44.28, 68.48)	1.78 (1.67, 1.89)
95+	0.02 (0.02, 0.03)	36.88 (30.00, 43.75)	0.32 (0.23, 0.39)	71.23 (52.46, 88.26)	2.07 (1.92, 2.22)

*Note*: Age‐specific incidence rate in each age group.

Abbreviations: ASIR, age‐standardized incidence rate; *CI*, confidence interval; EAPC, estimated annual percentage change; *UI*, uncertainty interval.

^a^ Incident cases reported as numbers (95% *UI*), per 1000.

^b^ ASIR reported as rates (95% *UI*), per 100,000.

^c^ EAPC in ASIR reported as estimates (95% *CI*).

**TABLE 2:** The number of deaths and the age‐standardized mortality rates of breast cancer in China in 1990 and 2019, and the estimated annual percentage changes from 1990 to 2019

Characteristics	1990		2019		1990–2019
	Deaths^a^	ASMR^b^	Deaths^a^	ASMR^b^	EAPC in ASMR^c^
Overall	41.80 (34.55, 49.51)	4.74 (3.96, 5.57)	96.31 (77.32, 118.09)	4.85 (3.91, 5.92)	0.06 (0.00, 0.12)
Sex					
Male	0.37 (0.30, 0.45)	0.10 (0.08, 0.12)	2.81 (2.15, 3.53)	0.29 (0.22, 0.36)	6.21 (5.21, 7.22)
Female	41.43 (34.15, 49.15)	9.16 (7.61, 10.82)	93.50 (74.51, 115.42)	9.02 (7.19, 11.10)	−0.14 (−0.21, −0.06)
Age group^d^					
15–19	0.07 (0.06, 0.08)	0.05 (0.05, 0.07)	0.03 (0.02, 0.03)	0.03 (0.03, 0.04)	−2.15 (−2.43, −1.87)
20–24	0.16 (0.13, 0.21)	0.12 (0.10, 0.16)	0.08 (0.06, 0.10)	0.10 (0.08, 0.13)	−1.64 (−2.22, −1.05)
25–29	0.39 (0.29, 0.50)	0.35 (0.27, 0.45)	0.37 (0.28, 0.49)	0.34 (0.25, 0.44)	−0.80 (−1.56, −0.04)
30–34	1.12 (0.87, 1.41)	1.26 (0.98, 1.60)	1.48 (1.13, 1.89)	1.15 (0.87, 1.47)	−1.15 (−1.81, −0.48)
35–39	3.04 (2.39, 3.75)	3.32 (2.61, 4.10)	2.56 (1.94, 3.26)	2.53 (1.92, 3.23)	−1.40 (−1.62, −1.18)
40–44	3.77 (2.99, 4.59)	5.61 (4.44, 6.82)	4.39 (3.41, 5.56)	4.32 (3.35, 5.47)	−1.24 (−1.38, −1.10)
45–49	4.10 (3.26, 4.98)	7.94 (6.31, 9.63)	7.29 (5.69, 9.22)	6.00 (4.69, 7.60)	−1.53 (−1.95, −1.12)
50–54	5.52 (4.43, 6.66)	11.54 (9.26, 13.93)	12.31 (9.68, 15.35)	9.84 (7.74, 12.27)	−0.64 (−0.82, −0.46)
55–59	6.30 (5.12, 7.61)	14.50 (11.77, 17.51)	12.71 (10.04, 16.01)	13.40 (10.59, 16.88)	0.09 (−0.28, 0.46)
60–64	5.01 (4.13, 5.96)	14.15 (11.67, 16.81)	12.03 (9.63, 14.84)	15.31 (12.26, 18.89)	0.59 (0.40, 0.78)
65–69	4.21 (3.51, 4.99)	15.40 (12.83, 18.22)	13.02 (10.66, 15.81)	18.50 (15.15, 22.47)	0.82 (0.66, 0.98)
70–74	3.32 (2.82, 3.95)	17.59 (14.97, 20.94)	10.59 (8.65, 12.82)	22.14 (18.07, 26.79)	0.89 (0.81, 0.97)
75–79	2.42 (2.07, 2.81)	21.20 (18.17, 24.63)	8.05 (6.62, 9.73)	26.96 (22.20, 32.60)	0.92 (0.80, 1.04)
80–84	1.49 (1.27, 1.72)	26.41 (22.45, 30.53)	6.14 (4.98, 7.36)	32.22 (26.10, 38.58)	0.81 (0.69, 0.92)
85–89	0.66 (0.56, 0.77)	34.30 (29.00, 40.11)	3.37 (2.64, 4.01)	39.60 (30.99, 47.17)	0.45 (0.35, 0.55)
90–94	0.18 (0.15, 0.22)	49.10 (40.92, 58.13)	1.52 (1.16, 1.83)	67.66 (51.83, 81.36)	1.07 (0.96, 1.18)
95+	0.04 (0.03, 0.04)	58.3 (46.99, 69.29)	0.38 (0.28, 0.46)	84.62 (63.46, 102.62)	0.93 (0.74, 1.12)

*Note*: Abbreviations: ASMR, age‐standardized mortality rate; *CI*, confidence interval; EAPC, estimated annual percentage change; *UI*, uncertainty interval.

^a^ Deaths reported as numbers (95% *UI*), per 1000.

^b^ ASMR reported as rates (95% *UI*), per 100,000.

^c^ EAPC in ASMR reported as estimates (95% *CI*).

^d^ Age‐specific incidence rate in each age group.

**TABLE 3:** The number of DALYs and the age‐standardized DALYs rates of breast cancer in China in 1990 and 2019, and the estimated annual percentage changes from 1990 to 2019

Characteristics	1990		2019		1990–2019
	DALYs^a^	ASDR^b^	DALYs^a^	ASDR^b^	EAPC in ASDR^c^
Overall	1435.10 (1184.14, 1708.68)	145.67 (121.08, 172.57)	2957.45 (2408.51, 3590.17)	144.15 (117.26, 174.99)	−0.13 (−0.19, −0.06)
Sex					
Male	11.62 (9.46, 14.00)	2.45 (2.00, 2.93)	80.21 (61.44, 100.88)	7.73 (5.98, 9.62)	6.59 (5.51, 7.67)
Female	1423.49 (1173.66, 1696.00)	294.04 (243.51, 349.12)	2877.24 (2323.69, 3513.54)	277.98 (224.35, 339.93)	−0.35 (−0.43, −0.26)
Age group^d^					
15–19	5.13 (4.33, 6.15)	4.04 (3.42, 4.85)	2.01 (1.68, 2.39)	2.67 (2.24, 3.19)	−1.94 (−2.22, −1.66)
20–24	11.40 (9.11, 14.23)	8.61 (6.88, 10.75)	6.00 (4.67, 7.50)	7.32 (5.70, 9.16)	−1.41 (−1.98, −0.83)
25–29	24.69 (19.12, 31.37)	22.40 (17.35, 28.46)	25.56 (19.63, 32.58)	23.09 (17.73, 29.42)	−0.57 (−1.31, 0.18)
30–34	65.34 (51.69, 82.11)	73.82 (58.40, 92.76)	91.93 (71.58, 114.84)	71.21 (55.45, 88.96)	−0.94 (−1.60, −0.29)
35–39	162.39 (128.71, 200.20)	177.46 (140.66, 218.78)	144.16 (111.74, 179.68)	142.88 (110.75, 178.09)	−1.20 (−1.42, −0.99)
40–44	182.89 (145.82, 222.20)	271.98 (216.86, 330.44)	226.01 (178.04, 277.39)	222.35 (175.16, 272.90)	−1.01 (−1.15, −0.88)
45–49	178.30 (143.30, 215.90)	344.75 (277.07, 417.44)	335.75 (267.68, 417.24)	276.65 (220.56, 343.79)	−1.31 (−1.72, −0.91)
50–54	212.04 (171.62, 254.08)	443.58 (359.02, 531.52)	496.74 (398.21, 615.73)	397.07 (318.31, 492.17)	−0.46 (−0.63, −0.28)
55–59	212.14 (173.84, 254.47)	488.11 (399.98, 585.49)	448.08 (360.94, 552.47)	472.46 (380.58, 582.54)	0.27 (−0.10, 0.65)
60–64	145.86 (121.82, 172.56)	411.79 (343.93, 487.18)	367.44 (300.45, 444.69)	467.74 (382.46, 566.08)	0.78 (0.58, 0.98)
65–69	103.33 (86.91, 121.93)	377.58 (317.58, 445.51)	334.98 (278.89, 400.42)	475.93 (396.24, 568.90)	1.01 (0.85, 1.18)
70–74	66.76 (56.99, 79.09)	354.04 (302.23, 419.42)	222.10 (184.31, 264.45)	464.10 (385.13, 552.59)	1.05 (0.97, 1.13)
75–79	38.66 (33.41, 44.68)	338.79 (292.77, 391.58)	132.74 (110.95, 157.29)	444.75 (371.74, 527.01)	1.05 (0.92, 1.17)
80–84	18.40 (15.63, 21.18)	326.35 (277.17, 375.63)	78.22 (64.04, 92.37)	410.24 (335.86, 484.44)	0.93 (0.81, 1.06)
85–89	6.23 (5.31, 7.27)	324.90 (276.88, 379.18)	32.56 (25.77, 38.36)	382.81 (303.00, 451.00)	0.55 (0.45, 0.64)
90–94	1.34 (1.12, 1.58)	359.64 (300.48, 423.12)	11.09 (8.56, 13.15)	494.00 (381.26, 585.98)	1.08 (0.97, 1.19)
95+	0.21 (0.17, 0.24)	332.14 (268.74, 392.11)	2.09 (1.58, 2.53)	468.07 (354.68, 566.71)	0.85 (0.67, 1.03)

*Note*: Abbreviations: ASDR, age‐standardized DALY rate; *CI*, confidence interval; DALYs, disability‐adjusted life‐years; EAPC, estimated annual percentage change; *UI*, uncertainty interval.

^a^ DALYs reported as numbers (95% *UI*), per 1000.

^b^ ASDR reported as rates (95% *UI*), per 100,000.

^c^ EAPC in ASDR reported as estimates (95% *CI*).

^d^ Age‐specific incidence rate in each age group.

**FIGURE 2:**
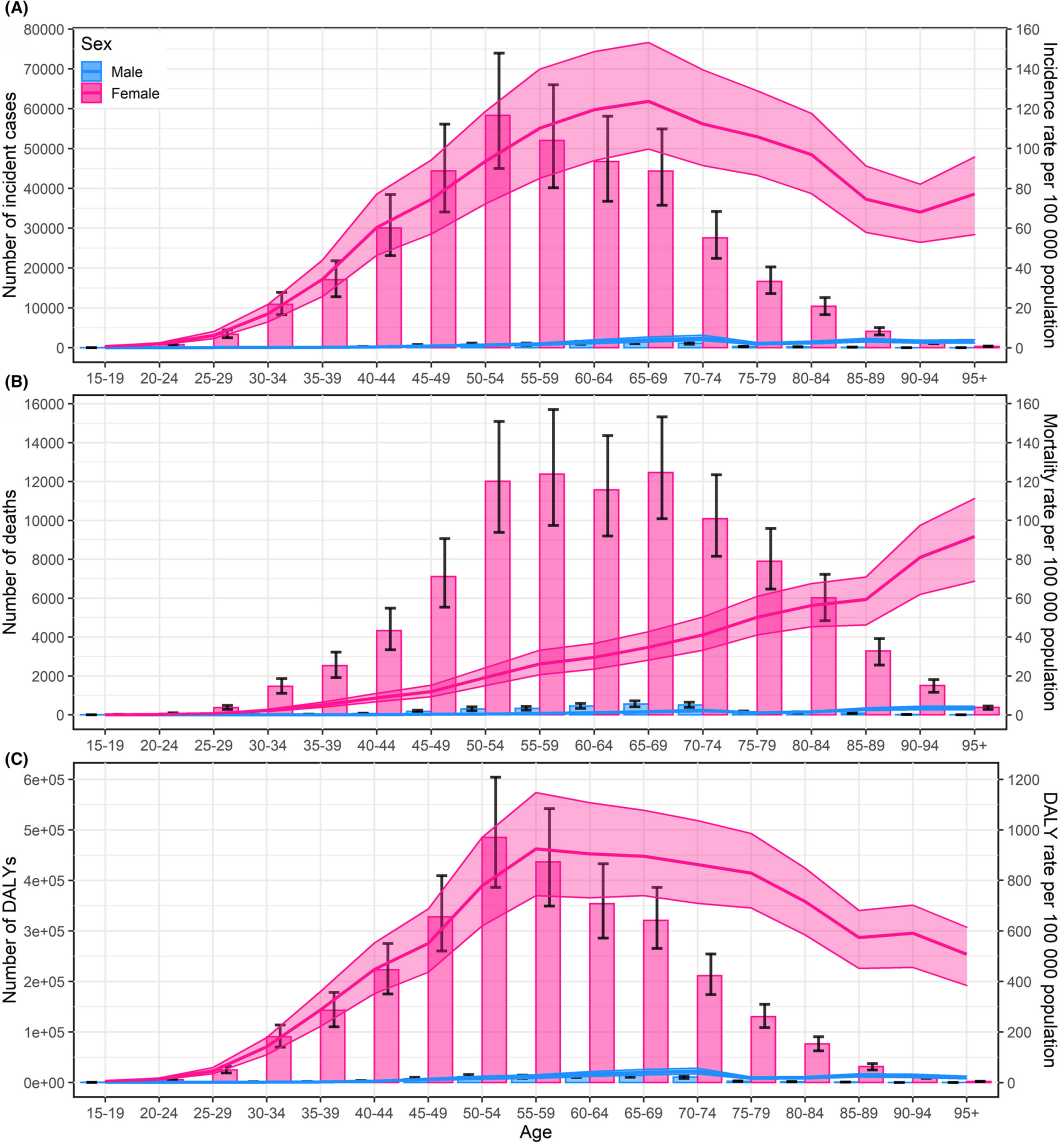
The trend in number and rates of breast cancer incidence (A), mortality (B), and DALYs (C) by age and sex in China, 2019. The bar graphs represent the observed number and the error bars indicate the 95% uncertainty intervals (*UI*s); the line charts represent the age‐specific rates and the shading indicates the 95% *UI*s. DALY, disability‐adjusted life‐years. The male information is also separately shown in Supplementary Materials.

In 2019, the number of breast cancer burden increased first and then decreased with age, which mainly occurred in individuals of 50–69 years old. For both sexes combined, the highest age‐specific rates of incidence (71.23/100,000; 95% *UI*: 52.46, 88.26) and mortality (84.62/100,000; 95% *UI*: 63.46, 102.62) were observed in 95+ age group, and the highest age‐specific DALY rate (494.00/100,000; 95% *UI*: 381.26, 585.98) was in 90–94 age group (Tables 1, 2, 3). From 1990 to 2019, the age‐specific incidence rates increased with time for all age groups, but for nearly all individuals under the age of 55, the age‐specific rates of mortality and DALY showed a slight decline (Tables 1, 2, 3; Figures S4–S6). The age‐specific rates in females were substantially higher than that in males. As for females, the age‐specific incidence and DALY rates reach the maximum approximately in individuals aged 55–69, while the age‐specific mortality rates increased monotonically with age (Figure 2).

### 3.2 The geographical breast cancer burden in China

**FIGURE 3:**
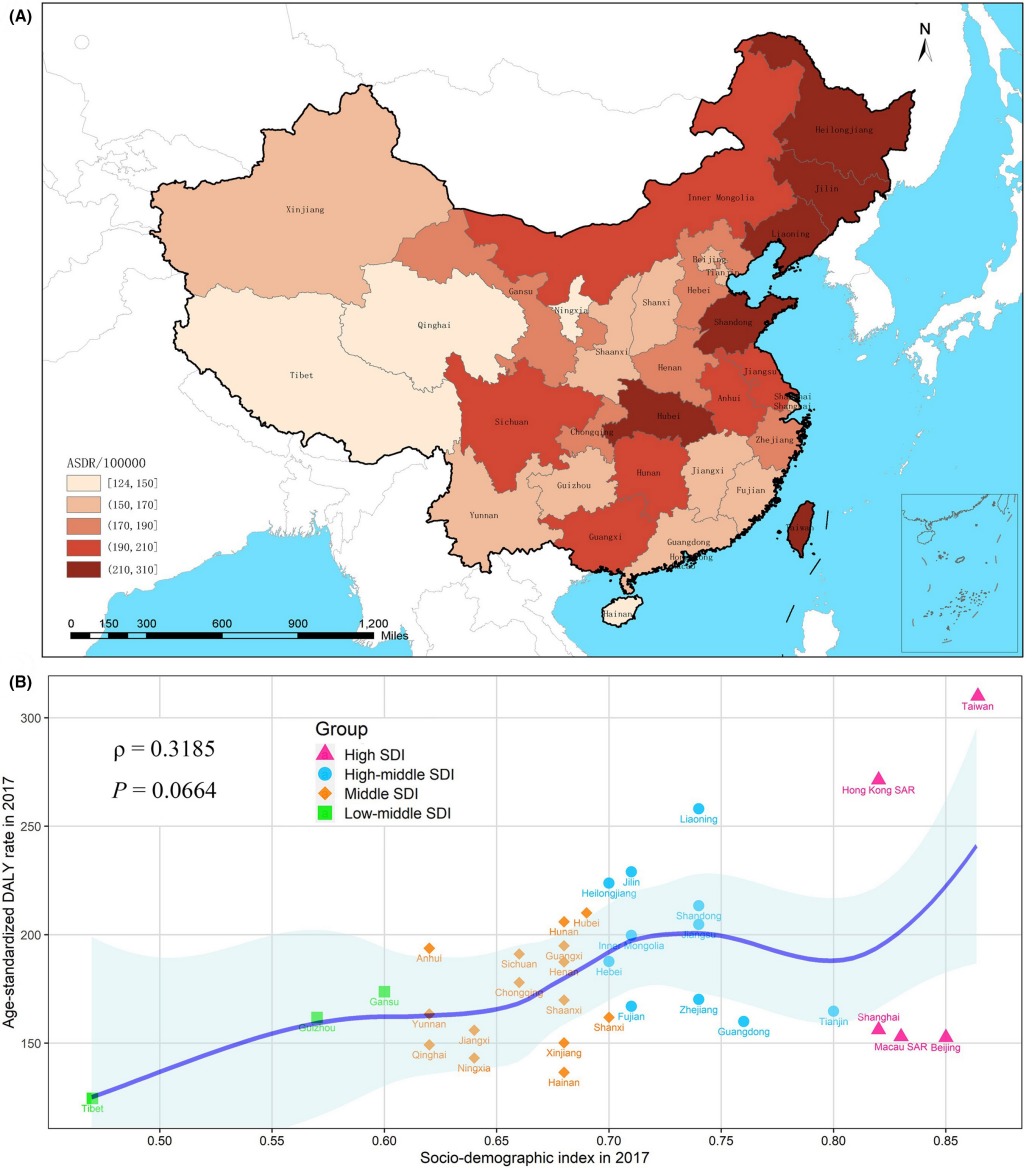
The subnational trend in breast cancer DALYs in China, 2017. (A) The age‐standardized DALY rate of breast cancer in 34 provinces of China. (B) The association between age‐standardized DALY rate (per 100,000) and Socio‐demographic Index values in 34 provinces of China. DALY, disability‐adjusted life‐year.

Figure 3 presented the geographical distribution of breast cancer DALYs in 2017 in China. ASDR varied significantly across the country. Taiwan (309.99 per 100,000 persons), Hong Kong SAR (271.38 per 100,000 persons), Liaoning (258.01 per 100,000 persons), Jilin (228.93 per 100,000 persons), Heilongjiang (223.79 per 100,000 persons), Shandong (213.36 per 100,000 persons), and Hubei (210.05 per 100,000 persons) were the seven provinces of the highest ASDR, while Tibet (124.54 per 100,000 persons), Hainan (136.53 per 100,000 persons), Ningxia (143.20 per 100,000 persons), and Qinghai (149.26 per 100,000 persons) were the four provinces of the lowest (Figure 3A). China was a high‐middle‐SDI country in 2017, with the SDI value of 0.71, 34 provinces of which varied from the low‐middle to high SDI quintiles, with the SDI values ranging from 0.47 (Tibet) to 0.85 (Beijing). A slight positive association was observed between ASDR with SDI values (*ρ* = −0.319, *p* = 0.066), although ASDR in some high‐SDI provinces, including Beijing (152.65/100,000), Macau SAR (152.99/100,000) and Shanghai (156.19/100,000) were much lower than expected (Figure 3B).

### 3.3 The temporal trend in DALYs attributable to risk factors

**FIGURE 4:**
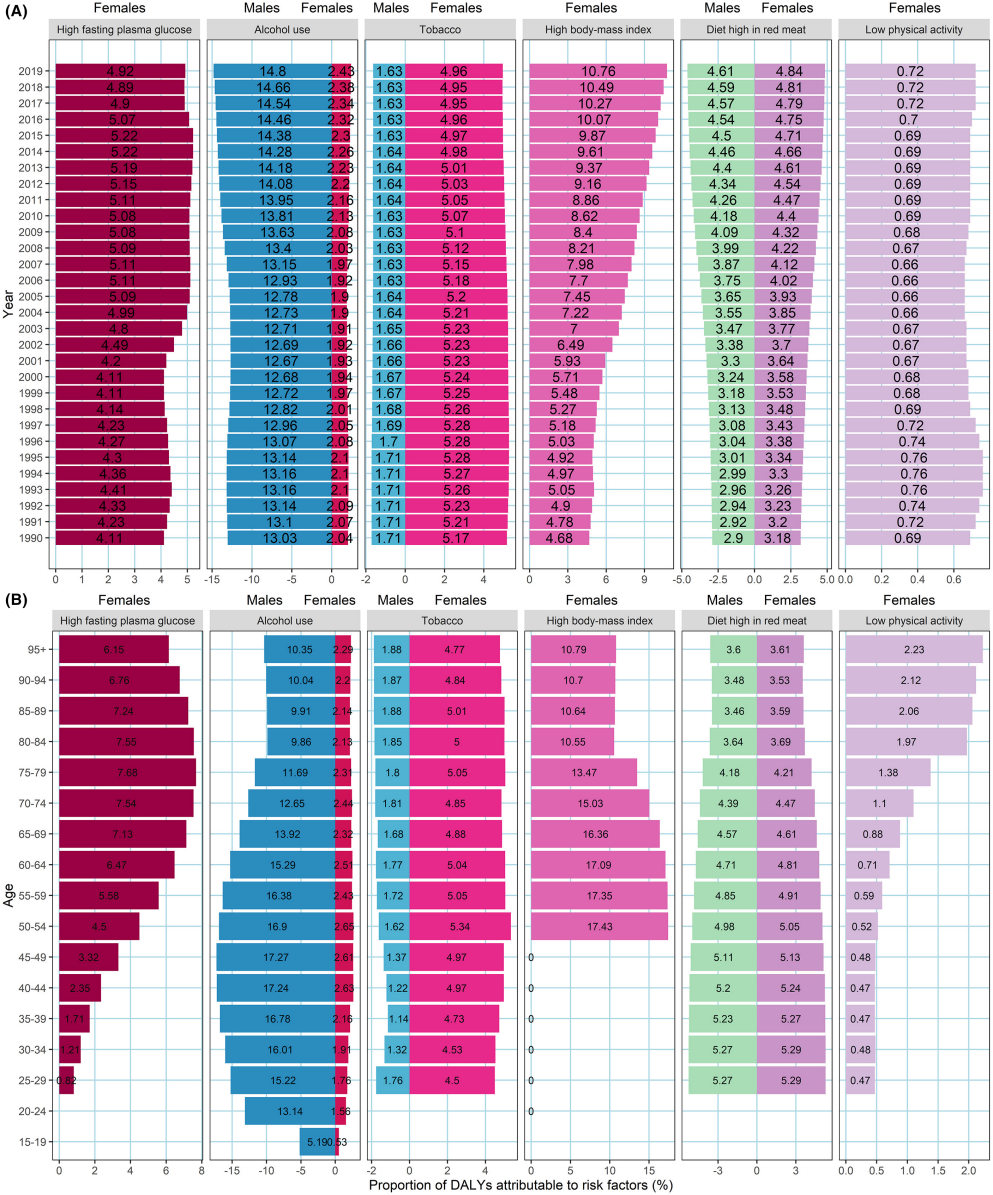
The temporal trend in proportion of breast cancer DALYs attributable to risk factors by sex, from 1990 to 2019 (A) and for different age groups in 2019 (B). DALY, disability‐adjusted life‐year.

Figure 4A showed the variation in the proportion of DALYs attributable to the selected six risk factors with sex and year, in which only alcohol use, tobacco (secondhand smoke), and diet high in red meat were included for males. From 1990 to 2019, the proportion of DALYs attributable to tobacco (5.15%–4.87%) decreased slightly, but the proportions of DALYs attributable to the other five risk factors were all in upward trends for both sexes (data not shown; https://vizhub.healthdata.org/gbd‐results/). Tobacco (5.17%) and high BMI (4.68%) were the major contribution to female breast cancer DALYs in 1990, whereas high BMI (10.76%) became the first contribution in 2019, following by tobacco (4.96%), high fasting plasma glucose (4.92%) and diet high in red meat (4.84%). For male breast cancer DALYs, alcohol use was always the first contribution (12.67%–14.80%) from 1990 to 2019.

In 2019, the proportion of DALYs attributable to these risk factors also varied with sex and age (Figure 4B). For both sexes, the proportion of DALYs attributable to diet high in red meat decreased with age (5.29%–3.53%), while the proportion of DALYs attributable to alcohol use and tobacco had little change with age. For females, the proportion of DALYs attributable to high fasting plasma glucose and low physical activity increased significantly from less than 1% to 7.68% and 2.23%, respectively, but the proportion of DALYs attributable to high BMI decreased from 17.43% in 50–54 age group to approximately 10.59% in individuals of 85+ years old. Alcohol use was the first contribution to male breast cancer DALYs for all age groups, the proportion of which was 5–6 times higher than that in females (Figure 4B; Figure S7).

### 3.4 Projections of breast cancer incidence and mortality, 2020–2030

**FIGURE 5:**
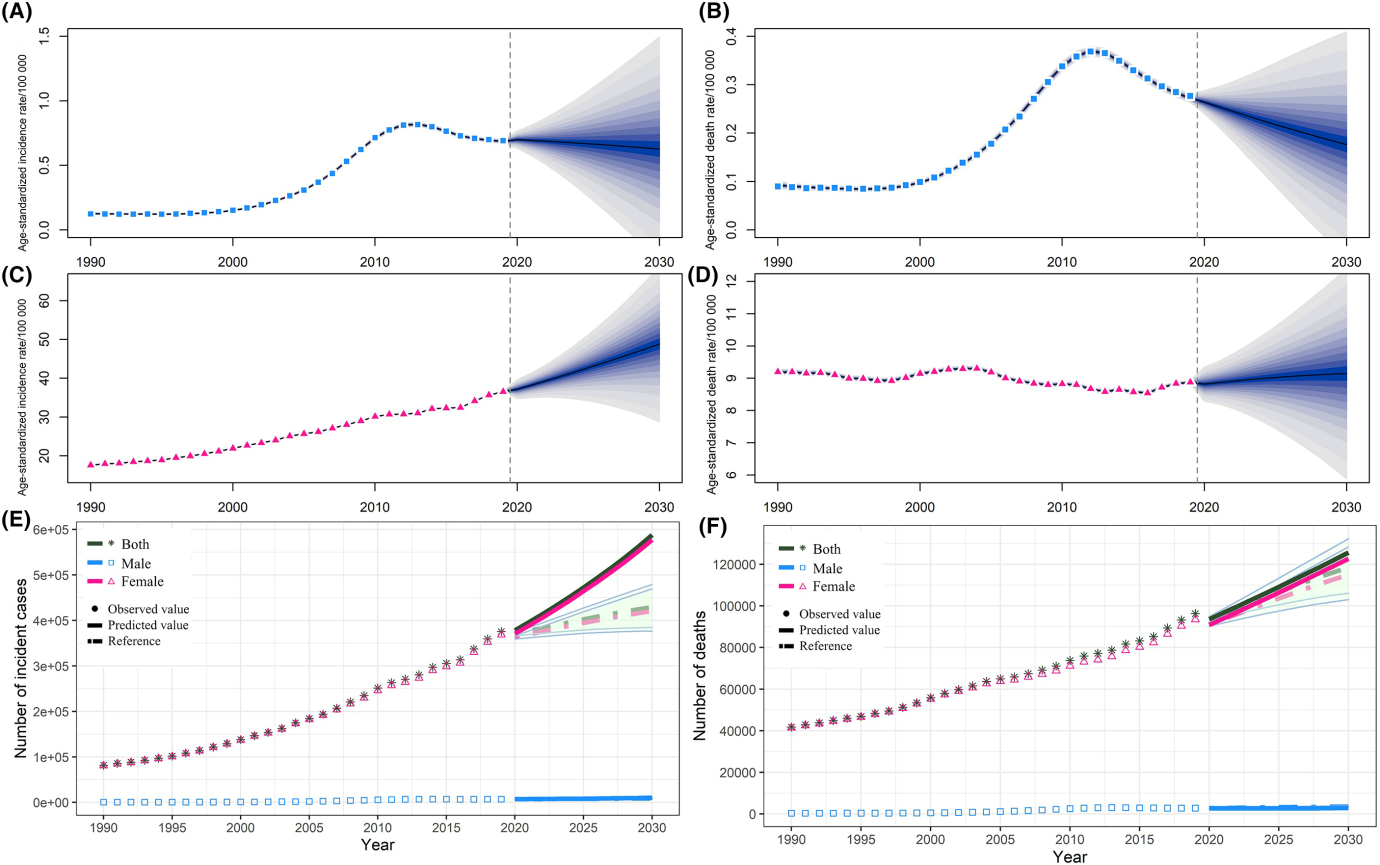
The projection of breast cancer incidence and mortality from 2020 to 2030 in China. (A) Age‐standardized incidence rate for male. (B) Age‐standardized mortality rate for male. (C) Age‐standardized incidence rate for female. (D) Age‐standardized mortality rate for female. (E) The projected number of incident cases; (F) The projected number of deaths. The dots present the observed values. The solid lines present the projected values. The dash‐and‐dot lines and light‐green shading present the reference calculated based on the observed values in 2019.

The projections of breast cancer incident cases, deaths and corresponding ASRs for 2020–2030 were presented in Figure 5. From 2020 to 2030, ASIR and ASMR of male breast cancer would decrease, and the ASDR showed a more noticeable decline than the ASIR (Figure 5A–B). However, as for female breast cancer, there would be a slightly upward trend in ASIR, and the ASDR remained relatively stable (Figure 5C–D). The projected number of breast cancer incident cases and deaths would increase steadily for both sexes, although the increment was much larger in females than that in males. Besides, our projections of breast cancer incident cases were substantially higher than the references with a 1% changing rate annually, while the projections of breast cancer deaths were within the corresponding references. In 2030, there would be 577,145 breast cancer incident cases and 122,674 deaths in females, and 10,585 incident cases, and 2949 deaths in males (Figure 5E–F).

## 4 DISCUSSION

In this analysis, we presented the spatiotemporal trends in breast cancer burden along with six attributable risk factors from 1990 to 2030 in China. The results suggested that breast cancer was still a major health burden for China, with 375,484 incident cases, 96,306 deaths, and 2,957,454 DALYs occurring in 2019, and the absolute burden of breast cancer would continue to increase until 2030. Breast cancer DALYs varied with the provincial SDI values, with the higher ASDR mainly observed in high and high‐middle SDI provinces. High BMI was the first contribution to breast cancer DALYs for females, and alcohol use for males.

China always had the largest absolute burden of breast cancer worldwide from 1990 to 2019.^17^ Our analysis found that the number of breast cancer incident cases, deaths, and DALYs increased obviously in China during this period, which could be due to the rapid population growth.^26^, ^27^, ^28^ There was a population of 1.1 billion in 1990, and 1.4 billion in 2019, with an annual natural growth rate of 7.74‰ in China,^29^ and thus the absolute burden of breast cancer would increase significantly, even though there was only a slight change of relative ratio. The enormous population base would bring the country great disease epidemics and burden of breast cancer.

A continuous increase in ASIR was found from 1990 to 2019, especially for people aged 55–85 years. China was always in an increasing aging society since early 1980s,^30^ which exacerbated the breast cancer burden significantly for the elderly. On the other hand, westernization in China was also an important reason for the aggravation of breast cancer, which referred to the westernization of socioeconomic backgrounds and lifestyle.^31^ With the rapid development of society and economy, women tended to delay childbearing, even have fewer deliveries, and reduce breast‐feeding, thereby spending more time plunging the society. The lifestyle westernization was related to diet, obesity, and exercise,^32^ and resulted in the incidence rates in China increasing and approaching that of the western countries.

We also found that the breast cancer ASMR and ASDR showed a slight change, or even decreased during this period, especially for individuals aged 40–59. In China, female breast cancer screening guidelines suggested that females aged 45–70 should be screened and take an ultrasound test of breast cancer every 1 to 2 years.^33^ The increasing coverage of breast cancer screening contributed to the increased diagnosis of small cancers, and detection and treatment of early breast cancer,^34^ which was beneficial to the decreased mortality and DALYs of breast cancer in the long term. However, the screening programs have not been spread nationwide, especially in some low‐SDI provinces, for the huge human population, unequal distribution of mammography equipment, and inadequate insurance coverage,^35^ and mainly carried out in some high‐ and high‐middle‐SDI provinces, including Beijing, Shanghai, Tianjin, and Macau SAR. Therefore, ASDR of breast cancer in these provinces were much lower than expected.

High BMI and high fasting plasma glucose were identified as two potential risk factors contributing to breast cancer DALYs. The proportion of DALYs attributable to high BMI increased obviously from 1990 to 2019, which had become the first contribution by 2019. According to the study of NCD Risk Factor Collaboration,^36^ the rise in mean BMI continued to accelerate, and there was the largest absolute increase in the number of individuals with overweight and obesity in East Asia. Increased BMI is associated with increased risk of breast cancer. Meanwhile, the patterns of diet and activity in China changed, such as the higher prevalence of diet high in red and processed foods, and low physical activity, which was also associated with the risk of breast cancer.^37^, ^38^, ^39^ Alcohol use and tobacco were also identified as the risk factors contributing to breast cancer DALYs,^40^, ^41^ of which alcohol use was the first contribution to DALYs for males in our analysis. Although the mechanisms of alcohol use and risk of breast cancer were not known clearly, there was a linear dose–response association in between.^42^

Male breast cancer, distinct from female breast cancer in the biological standpoint, was a rare and usually ignored disease.^6^ We found different epidemiological features between male breast cancer and female breast cancer,^43^ that male breast cancer showed more obvious trends than female breast cancer, although it only accounted for nearly 2% of all breast cancer cases in China. Meanwhile, risk factors contributing to male breast cancer were not identical to female breast cancer, such as the discrepancy of high BMI and alcohol use.^44^ However, management of male breast cancer mainly depended on studies of female breast cancer,^4^, ^5^ not considering the discrepancy in epidemics and biological factors. Therefore, in our analysis, we also demonstrated the specific disease burden of male breast cancer, in order to provide evidence for developing tailored management strategies among males.

However, several limitations should be taken into consideration when interpreting the results. First, the disease burden of breast cancer at the provincial level in China was only assessed based on GBD 2017 from a previous study,^19^ in lack of temporal data on DALYs and SDI values of each provincial administrative unit. Second, it was noteworthy that the current breast cancer burden might be underestimated in China. The National Central Cancer Registry of China was established from 2002, and the number of local registries increased to 501 by August 2018, which covered 387 million population. Breast cancer burden in China were estimated based on the high‐quality parts of total registries, which could only involve in about 10% of the total population.^45^ Third, there would be changes in population and the allocation of medical resources in China after the Covid‐19 pandemic, which might impact on the projection from 2020 to 2030. Finally, GBD 2019 did not provide the data on pathology and subtypes of breast cancer, as well as other established risk factors, such as post‐pregnancy^10^ and hormone‐replacement therapy,^11^ for which the further analysis were confined.

In conclusion, we demonstrated the spatiotemporal trend in breast cancer burden along with attributable risk factors in China from 1990 to 2019, and made projections until 2030. The number of breast cancer incident cases, deaths, and DALYs in China was in an upward trend over the past three decades, and would continue to increase in the next 11 years. The socioeconomic status and relevant risk factors of breast cancer varied with sex and time, based on which the precise control and prevention strategies should be specifically developed for males and females.

## AUTHOR CONTRIBUTIONS

All authors contributed to the study design. Jie Li and Jinjin Nie performed the data collection, analysis, and result visualization. Cui Chen, Lili Wang, and Zhen Zhang contributed to the interpretations of results. The first draft of the manuscript was written by Jie Li and all the other authors carried out the manuscript revising. All authors read and approved the final manuscript.

## CONFLICT OF INTEREST

The authors have no relevant financial or non‐financial interests to disclose.

## DATA AVAILABILITY

All data could be extracted from the online Global Health Data Exchange (GHDx) query tool (https://vizhub.healthdata.org/gbd‐results/).

## ETHICS APPROVAL AND CONSENT TO PARTICIPATE

The GBD 2019 study is a publicly available database and all data were anonymous. The study protocol was exempted by the Ethical Board Review of our institute.

## Supporting information


Appendix S1
