# Supplementary material for: Changes in the disease burden of breast cancer along with attributable risk factors in China from 1990 to 2019 and its projections: An analysis of the global burden of disease study 2019
Source: Cancer Med. 2022 Jul 3;12(2):1888–902. doi: 10.1002/cam4.5006 (PMC9883426; doi:10.1002/cam4.5006)
Supplement: Supplementary file 1 — Appendix S1 [file CAM4-12-1888-s001.doc]

**Changes in the disease burden of breast cancer along with** **attributable risk factors in China from 1990 to 2019 and its projections: an analysis of the Global Burden of Disease Study 2019**

Running title: Breast cancer burden in China from 1990 to 2030

**
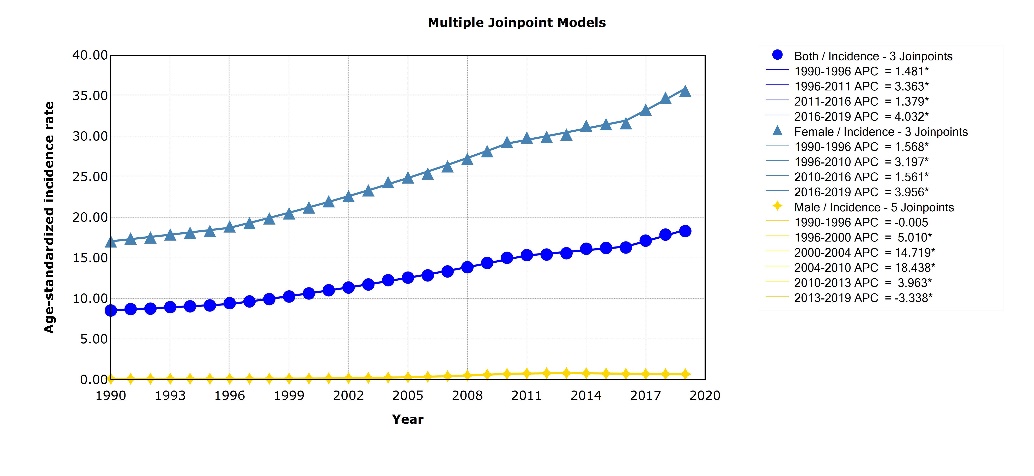
**

**Supplementary Figure S1.** The annual percentage changes in ASIR of breast cancer in China from 1990 to 2019. ASIR, age-standardized incidence rate.


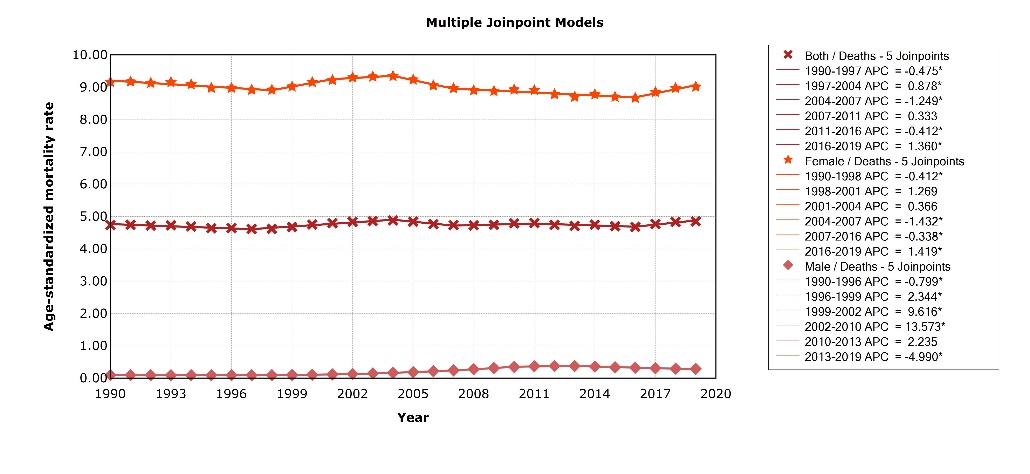


**Supplementary Figure S2.** The annual percentage changes in ASMR of breast cancer in China from 1990 to 2019. ASMR, age-standardized mortality rate.


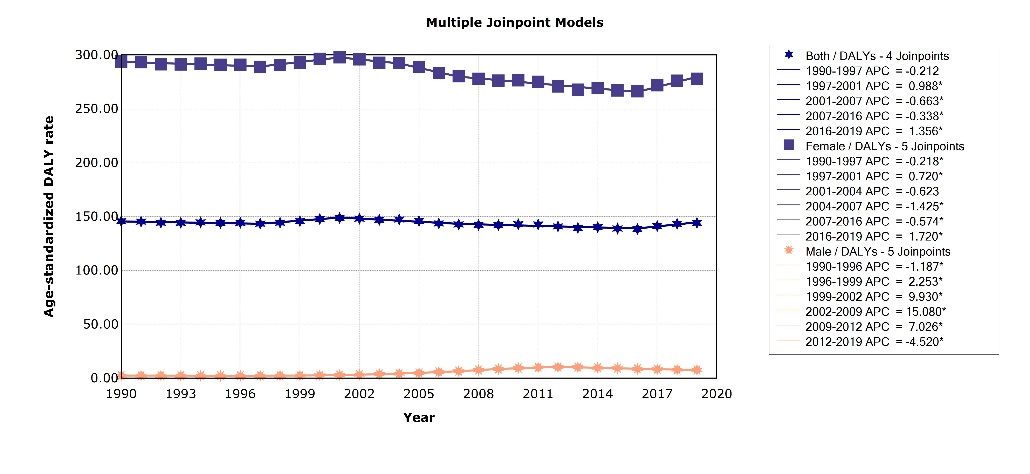


**Supplementary Figure S3.** The annual percentage changes in ASDR of breast cancer in China from 1990 to 2019. ASDR, age-standardized DALY rate; DALY, disability-adjusted life-year.

**Supplementary**
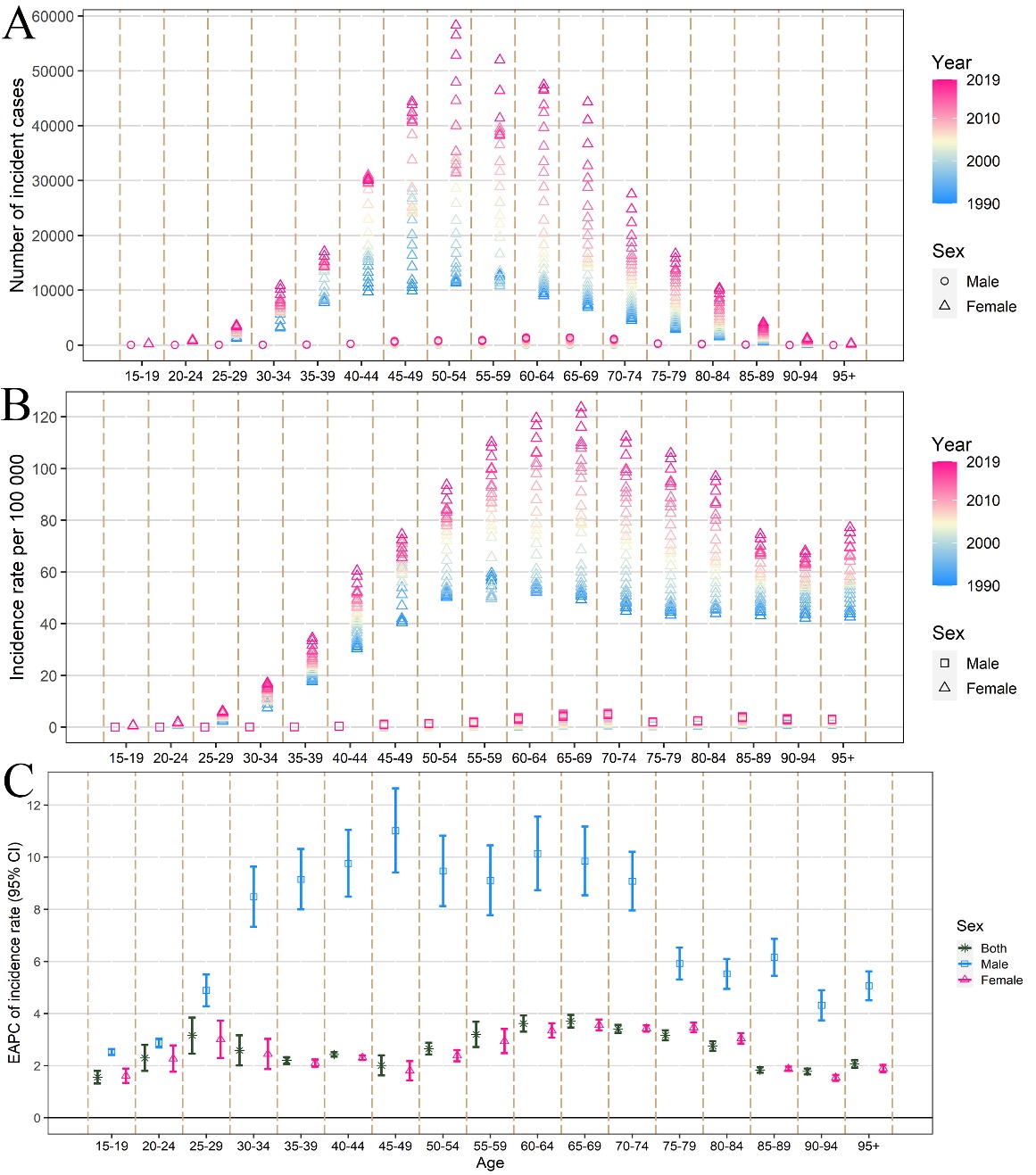
**Figure S4.** The patterns in number and rate of breast cancer incidence by sex and age group, from 1990 to 2019. (A) Number of incident cases by sex; (B) Age-specific incidence rate by sex; (C) EAPC in age-specific incidence rate by sex. *CI*, confidence interval; EAPC, the estimated annual percentage change.


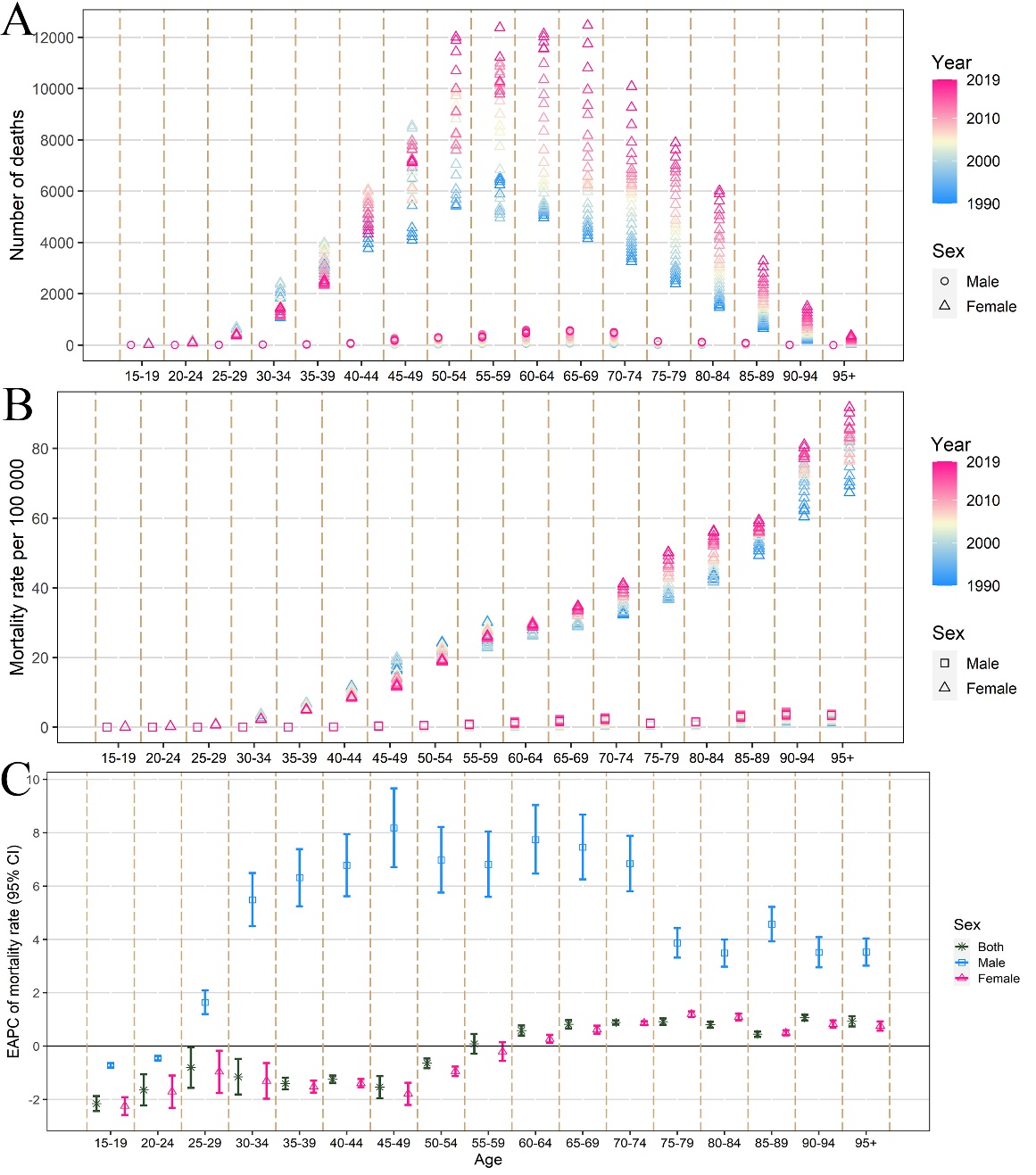
**Supplementary Figure S5.** The patterns in number and rate of breast cancer mortality by sex and age group, from 1990 to 2019. (A) Number of deaths by sex; (B) Age-specific mortality rate by sex; (C) EAPC in age-specific mortality rate by sex. *CI*, confidence interval; EAPC, the estimated annual percentage change.

**Supplementary Figure S6.** The patterns in number and rate of breast cancer DALYs by sex and age group, from 1990 to 2019. (A) Number of DALYs by sex; (B) Age-specific DALY rate by sex; (C) EAPC in age-specific DALY rate by sex. *CI*, confidence interval; DALY, disability-adjusted life-year; EAPC, the estimated annual percentage change
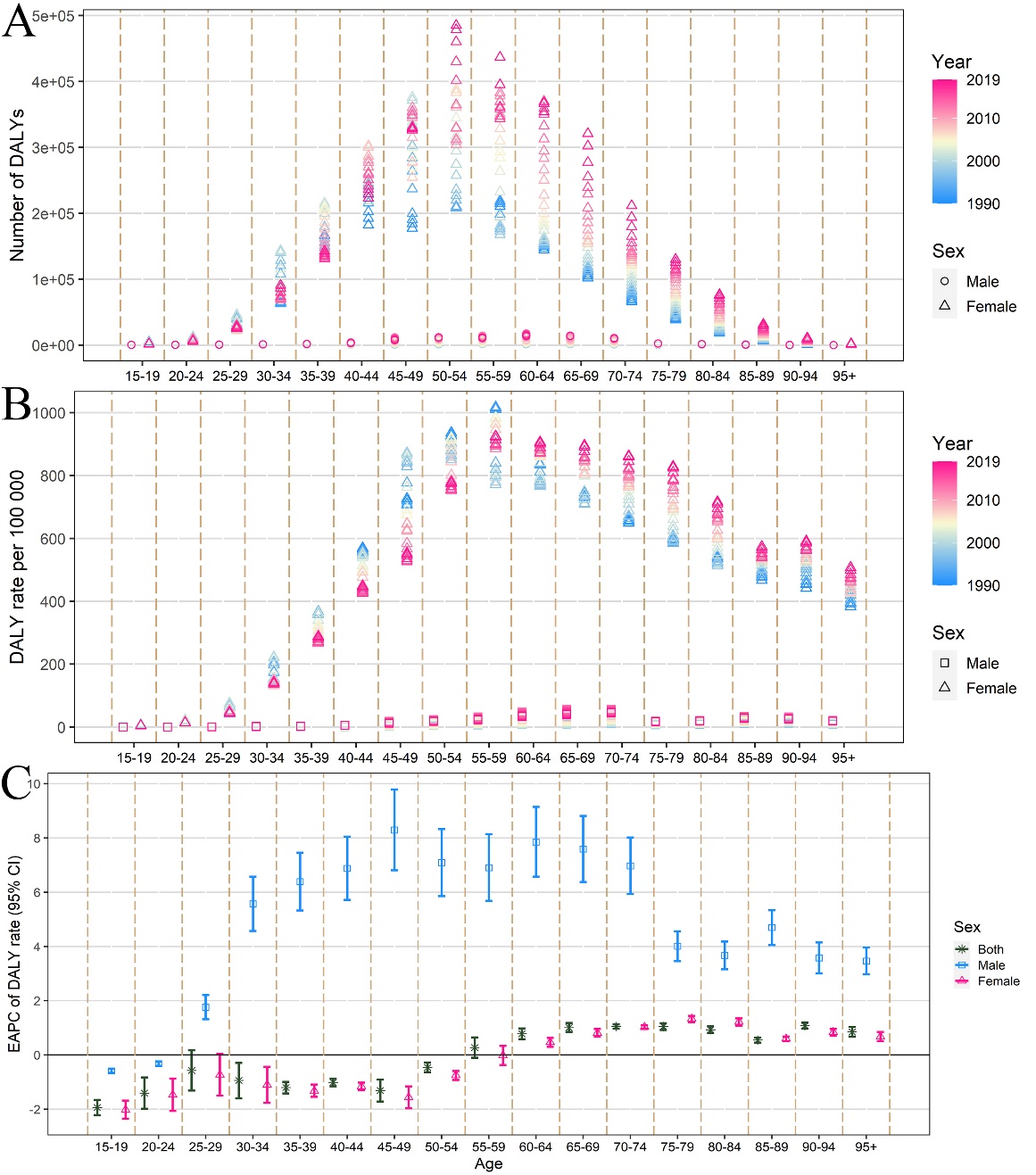
.

**Supplementary Figure S7.** The patterns in age-specific DALY rate of breast cancer attributable to risk factors by sex, from 1990 to 2019. DALY, disability-adjusted life-year
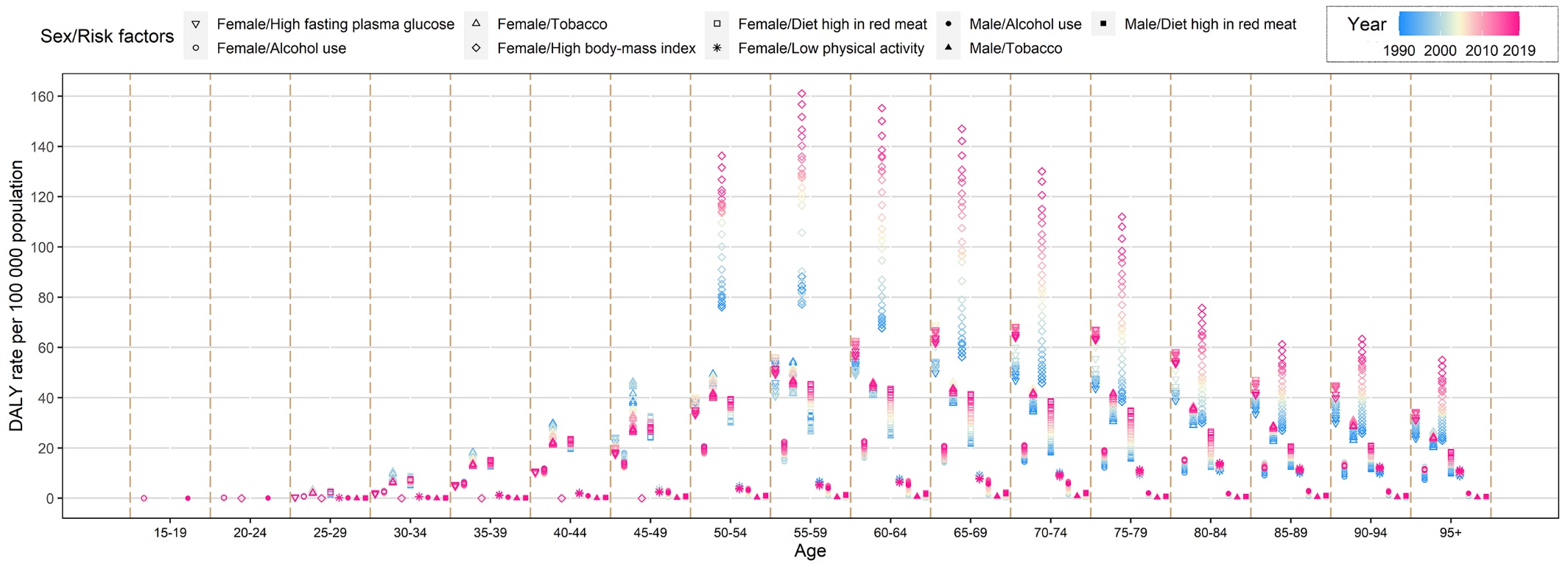
.

**Supplementary Figure S8.**
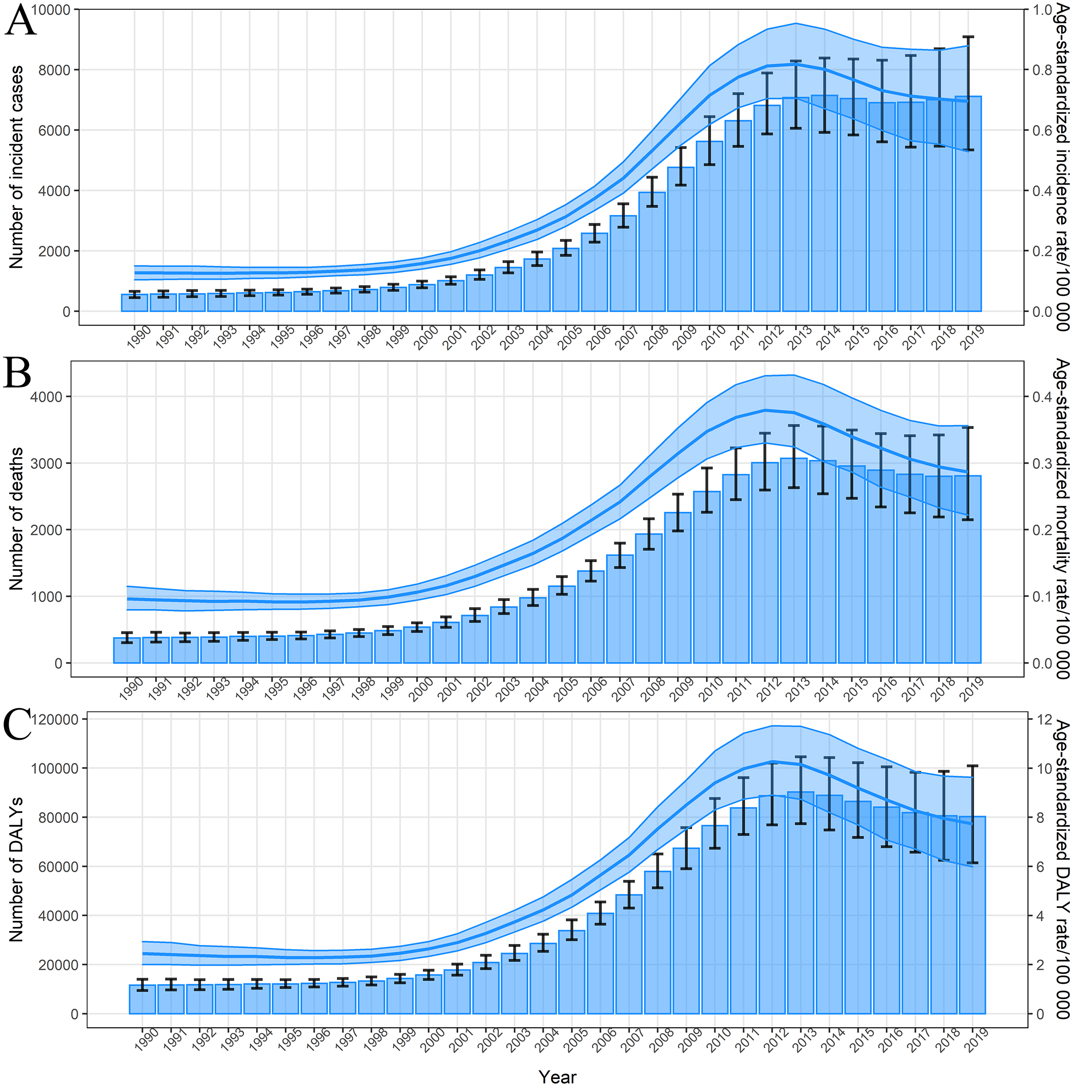
The temporal trend in number and ASRs of male breast cancer incidence (A), mortality (B), and DALYs (C) in China, 1990-2019. The bar graphs represent the observed number and the error bars indicate the 95% uncertainty intervals (*UI*s); the line charts represent the ASRs and the shading indicates the 95% *UI*s. ASR, age‐standardized rate; DALYs, disability-adjusted life-years.

**Supplementary Figure S9.**
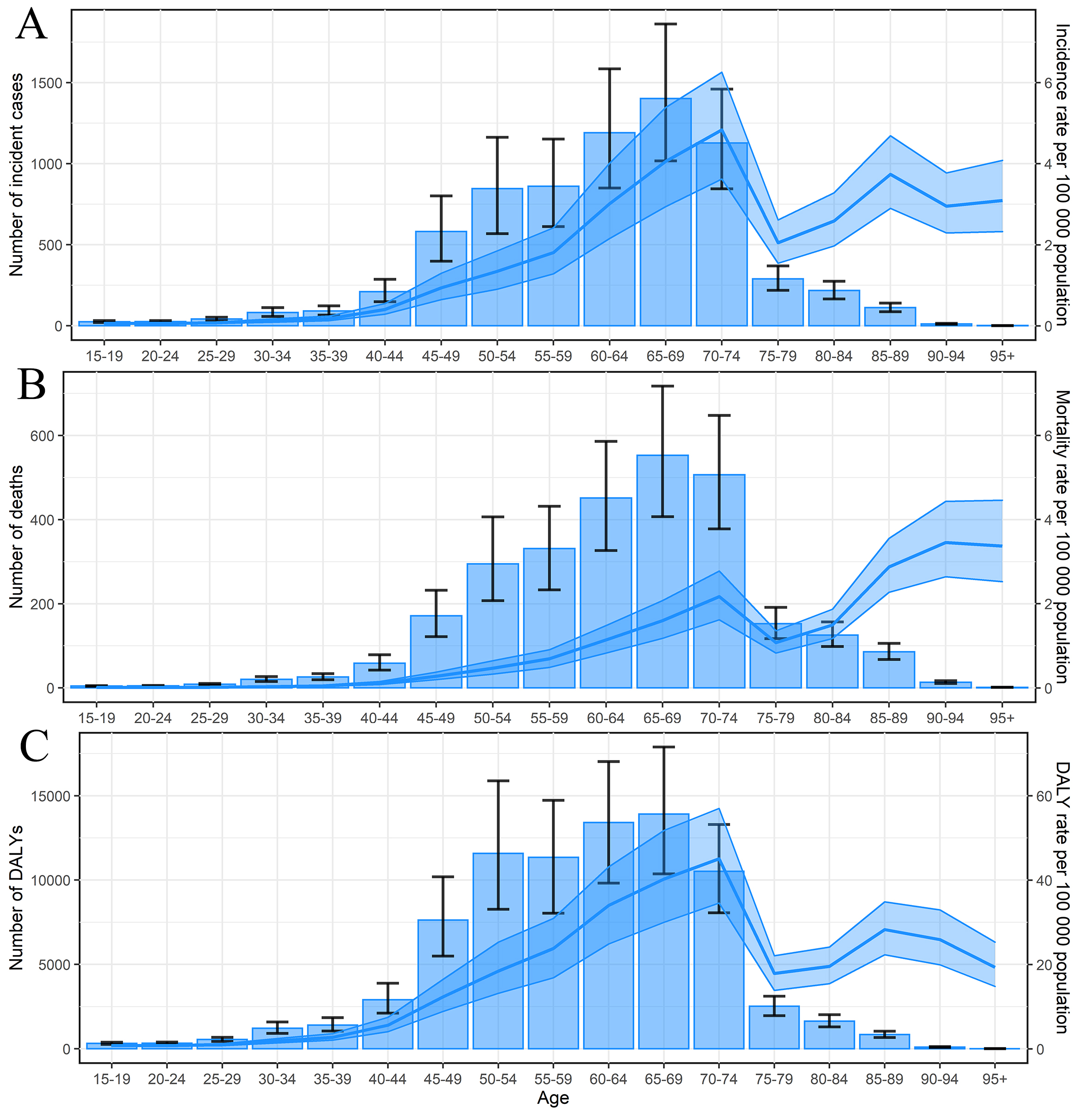
The trend in number and rates of male breast cancer incidence (A), mortality (B), and DALYs (C) by age in China, 2019. The bar graphs represent the observed number and the error bars indicate the 95% uncertainty intervals (UIs); the line charts represent the age-specific rates and the shading indicates the 95% UIs. DALY, disability-adjusted life-years.
